# Draft Genome Sequence of the Novonestmycin-Producing Strain Streptomyces sp. Z26, Isolated from Potato Rhizosphere in Morocco

**DOI:** 10.1128/MRA.01514-18

**Published:** 2019-01-03

**Authors:** Anina Buchmann, Carolina Cano-Prieto, Ahmed Nafis, Mustapha Barakate, Mohamed Baz, Lahcen Hassani, Nico Ortlieb, Timo H. J. Niedermeyer, Harald Gross

**Affiliations:** a Department of Pharmaceutical Biology, Pharmaceutical Institute, University of Tübingen, Tübingen, Germany; b German Centre for Infection Research (DZIF), Partner Site Tübingen, Tübingen, Germany; c Laboratory of Biology and Biotechnology of Microorganisms, Faculty of Sciences Semlalia, Cadi Ayyad University, Marrakech, Morocco; d Department of Microbiology and Biotechnology, Interfaculty Institute of Microbiology and Infection Medicine, University of Tübingen, Tübingen, Germany; e Department of Pharmaceutical Biology/Pharmacognosy, Institute of Pharmacy, University of Halle-Wittenberg, Halle (Saale), Germany; Loyola University Chicago

## Abstract

Streptomyces sp. strain Z26 exhibited antifungal activity and turned out to be a producer of the secondary metabolites novonestmycin A and B. The 6.5-Mb draft genome gives insight into the complete secondary metabolite production capacity and builds the basis to find and locate the biosynthetic gene cluster encoding the novonestmycins.

## ANNOUNCEMENT

As part of our ongoing efforts to investigate bioactive natural products from Actinobacteria (1–13), we isolated the strain Streptomyces sp. Z26 from potato rhizosphere in Morocco (14). It exhibited antibacterial as well as antifungal bioactivity (14), and a chemical analysis revealed that the strain produces the antifungal and cytotoxic natural products novonestmycins A and B (14, 15). In order to investigate the complete biosynthetic capacity for secondary metabolism and to locate, analyze, and prove the biosynthetic gene cluster of the novonestmycins, the sequencing of this strain was initiated.

Strain Z26 was cultivated for 4 days in 50 ml Trypticase soy broth (TSB) at 30°C under agitation (200 rpm). Genomic DNA (gDNA) extraction was performed using the Qiagen genomic-tip 100/G kit following the manufacturer’s protocol.

The genome of Z26 was sequenced using a combined Illumina/PacBio sequencing approach. Upon Nextera XT paired-end library preparation, gDNA was first subjected to 2 × 125-bp paired-end sequencing with the Illumina HiSeq 2500 system. FASTQ sequence files were generated using the Illumina Casava pipeline v1.8.3. The initial quality assessment was based on data passing the Illumina chastity filtering. Subsequently, reads containing the PhiX control signal were removed. In addition, reads containing (partial) adapters were clipped (up to a minimum read length of 50 bp). The second quality assessment was based on the remaining reads using the FastQC quality control tool v0.10.0. The final quality statistics included 3,393,302 reads and an average quality score (Phred) of 37. A *de novo* assembly was performed using CLC Genomics Workbench v7.0.4 to yield contigs.

In addition, a 10-kb genomic sublibrary of strain Z26 was produced and sequenced with PacBio single-molecule real-time (SMRT) technology. The data collected were processed and filtered using the SMRT Analysis software suite v2.3.0. The continuous long read (CLR) data were filtered by read length (>35 bp), subread length (>35 bp), and read quality (>0.75). The final quality statistics included 159,783 reads with an average read length of 5,122 bp and a maximum read length of 38,306 bp. The Illumina-based contigs were aligned against the PacBio CLR reads using BLASR v1 (16). Based on the alignment, contigs were placed into superscaffolds using the SSPACE-LongRead scaffolder v7.0.4 (17). The gapped regions within the superscaffolds were closed using GapFiller v1.10 (18). Software parameter settings were kept at the defaults, unless stated otherwise.

The final genome comprises three scaffolds with a total size of 6,501,581 bp and a G+C content of 73.4%. The assembled contigs were annotated with the NCBI Prokaryotic Genome Annotation Pipeline (PGAP) pipeline (19), yielding a total of 5,335 coding genes. Using autoMLST (http://automlst.ziemertlab.com), the overall genome analysis did not support the initial taxonomic classification of strain Z26 as S. phytohabitans (14); according to the average nucleotide identity (ANI) analysis, the closest related type strain species is S. pini NRRL-B-24728 (RefSeq assembly accession number GCF_900114215; ANI, 81.9%), while a multilocus sequence analysis revealed S. oceani SCSIO02100^T^ (RefSeq assembly accession number GCF_001751245) as the closest related species. Further bioinformatic analyses using antiSMASH v4.0.2 (20) revealed that strain Z26 possesses, besides the putative novonestmycin gene cluster, 20 orphan natural product biosynthetic gene clusters.

### Data availability.

This whole-genome sequencing (WGS) project has been deposited at DDBJ/ENA/GenBank under the accession number RCHV00000000. Raw sequencing data sets have been registered in the NCBI SRA database under the accession number SRP167030.
